# Improving the patient involvement in research and development on acute psychiatric wards – an audit and quality improvement project

**DOI:** 10.1192/bjo.2021.324

**Published:** 2021-06-18

**Authors:** Ioana Varvari, Hany El – Sayeh, Shona McIlrae, Susan Bonner

**Affiliations:** 1 Psychiatry Registrar, Tees, Esk and Wear Valleys NHS Foundation Trust; 2 Consultant Psychiatrist and Director of Medical Education,Tees, Esk and Wear Valleys NHS Foundation Trust; 3 Consultant Psychiatrist and Clinical Director,Tees, Esk and Wear Valleys NHS Foundation Trust; 4 Research nurse, Tees Esk and Wear Valleys NHS foundation Trust

## Abstract

**Aims:**

The local audit aimed at measuring awareness of research and development policies and implementation of local and national standards. Our findings generated a quality improvement project with two main objectives: first, improving patient approach and recruitment in research and second, improving trainee satisfaction within our trust.

**Method:**

A cohort of new inpatient admissions was identified over a period of 4 weeks, between October 2019 and November 2019, on the two psychiatric wards at the Briary Wing, Harrogate District Hospital. Based on local and national standards, we designed and developed a qualitative (questionnaire) and quantitative (audit tool) approach that was aimed at both staff and patients. Our steps included: assessing awareness and implementation of standards, a retrospective collection of data on the wards, and analysis of the data in Microsoft Excel.

**Result:**

Only one ward implemented the local guidance from which we identified a sample of 14 consecutive new admissions that were currently present on the ward and were able to answer our questions. 13 of those patients were noted as ‘approached’ on our visual board from which only 3 patients remembered reading a leaflet about research options in the admission pack, however, they have not been verbally informed. There was no process in place to assure the re-approaching of initially unwell patients or to follow up on discharge for those interested. Documentation was available in only 9 of the cases and was nonspecific: ‘admission pack done’.

**Conclusion:**

The awareness and understanding of Research and Development policies are poor and they are difficult to apply in practice in a busy inpatient environment without a clear process in place. This results in patients missing the opportunity to learn and understand more about research or to participate in ongoing studies. Quality improvement work needs to be done to improve patient recruitment in research in inpatient settings. Simple flow charts and stepwise processes as exemplified by our action plan have the potential to improve service quality, as well as patient and trainee satisfaction.

